# Electrophysiological evidence for changes in attentional orienting and selection in functional somatic symptoms

**DOI:** 10.1016/j.clinph.2018.09.027

**Published:** 2019-01

**Authors:** Maayan Karlinski, Alexander Jones, Bettina Forster

**Affiliations:** a Cognitive Neuroscience Research Unit, City, University of London, London, UK; b Department of Molecular Genetics, Weizmann Institute of Science, Rehovot, Israel; c Middlesex University, London, UK

**Keywords:** ERPs, Tactile, Attention, medically unexplained symptoms, Functional somatic symptoms, SDQ

## Abstract

**Objective:**

We investigated changes in attention mechanisms in people who report a high number of somatic symptoms which cannot be associated with a physical cause.

**Method:**

Based on scores on the Somatoform Disorder Questionnaire (SDQ-20; Nijenhuis et al., 1996) we compared two non-clinical groups, one with high symptoms on the SDQ-20 and a control group with low or no symptoms. We recorded EEG whilst participants performed an exogenous tactile attention task where they had to discriminate between tactile targets following a tactile cue to the same or opposite hand.

**Results:**

The neural marker of attentional orienting to the body, the Late Somatosensory Negativity (LSN), was diminished in the high symptoms group and attentional modulation of touch processing was prolonged at mid and enhanced at later latency stages in this group.

**Conclusion:**

These results confirm that attentional processes are altered in people with somatic symptoms, even in a non-clinical group. Furthermore, the observed pattern fits explanations of changes in prior beliefs or expectations leading to diminished amplitudes of the marker of attentional orienting to the body (i.e. the LSN) and enhanced attentional gain of touch processing.

**Significance:**

This study shows that high somatic symptoms are associated with neurocognitive attention changes.

## Highlights

• We investigated the electrocortical markers of attention in people with high functional somatic symptoms.
• Neural signature of attentional orienting was diminished while attentional selection was enhanced.
• Results show changes in attention processes which fit with predictive coding framework accounts.

## 1 Introduction

Medically unexplained symptoms (MUS) or functional somatic symptoms are characterised by unpleasant physical sensations with medical characteristics that do not correspond adequately to any acknowledged medical impairment. The nature of symptoms greatly varies, ranging from headaches, joint weaknesses, back pains, and heart palpitation, to severe cases of temporal blindness, motor paralysis, and even epileptic seizure. However, the underlying cause of MUS remains largely unknown (American Psychiatric Association, 2013, Hatcher and Arroll, 2008). While some researchers have suggested that the overall increase in pain sensitivity may be attributed to biochemical deficits and hormonal dysregulations in chronic MUS patients (Rief et al., 2004, Bohmelt et al., 2005), others have pointed out that psychological mechanisms need to be considered (Deary et al., 2007, Rief et al., 1998, Van den Bergh et al., 2017 for reviews). Furthermore, several cognitive models have proposed that chronic MUS are induced by a somatic attentional bias*.* That is, people who experience MUS might process, perceive, or interpret somatic information differently to people who do not suffer these symptoms (see Deary et al., 2007). These conditions are often referred to as *somatoform dissociation –* a dissociation between physical information and its bodily representation (Kienle et al., 2017, Nijenhuis, 2004, Ratcliffe and Newport, 2016).

Several cognitive models have been put forward trying to explain the underlying causes and symptoms of MUS. The *Somatosensory Amplification Model* (Barsky and Wyshak, 1990) has influenced several later models, which commonly suggest that MUS are provoked by an increased tendency to direct attention towards bodily sensation and the attribution of the sensations to serious illness. Alternatively, models like the *Signal Filtering Model* (Rief and Barsky, 2005) emphasise the importance of information filtering, suggesting that people who experience chronic MUS amplify ordinary bodily signals that usually do not reach consciousness. Similarly, more recent models also emphasis changes in attention mechanisms as mediators of MUS but explain these in the predictive framework (Edwards et al., 2012, Van den Bergh et al., 2017). While these types of models highlight changes of attention towards the body, surprisingly, only few studies have explored how people with chronic MUS attend to the sense of touch or internal body signals (e.g., heartbeat). Studies investigating the relationship between heartbeat detection ability and scores on the Somatosensory Amplification Scale (SASS; Barsky and Wyshak, 1990) have reported contradictory results with one study in support (Mailloux and Brener, 2002) but two studies (Aronson et al., 2001, Barsky and Borus, 1995) not finding any relationship between the SSAS score and ability to sustain attention to the heartbeat. On the other hand, Brown et al. (2007) investigated the relationship between self-report somatoform dissociation and attentional bias to touch induced by threatening body related pictures and found that these to be negatively correlated. Further, Brown et al. (2010) reported different effects of exogenous attention on tactile discrimination in participants with low and high scores on the Somatoform Dissociation Questionnaire (Nijenhuis et al., 1996). Together, these latter behavioural results support the notion that somatoform dissociation may be related to changes in attention to the body.

Neurophysiological studies in animals, and neuroimaging studies in humans have advanced our understanding of the neural mechanisms underlying selective attention in the somatosensory system (see Gomez-Raminez et al., 2016 for review). In particular, recent electrophysiological studies have shown that when focusing attention to the body, changes in the sensory specific neural responses are also observed. Exploring the effects of how we focus attention has typically been investigated in a cue-target paradigm (see Posner, 2016 for a review). A cue is used to voluntarily or reflexively direct attention to a spatial location. Participants then respond to a target presented at an attended or unattended location, thus providing a measure of shifts of attention. During the orienting of attention to a body location following the onset of the cue, a sequence of lateralized components are usually reported over anterior and central electrode sites (Forster et al., 2009, Gherri and Forster, 2012, Gherri et al., 2016, Jones and Forster, 2012). First, the Anterior Directing Attention Negativity (ADAN) is seen as an enhanced negativity contralateral to the attention directing cue elicited around 400–600 ms after cue onset. For example, a cue indicating a shift of attention to the left would typically result in a larger negativity over the right compared to the left hemisphere, This component has originally been associated with reflecting top-down control processes (Eimer et al., 2005, Hopf and Mangun, 2000, Nobre et al., 2000) but its exact functional significance is still under debate (see Green and McDonald, 2006, Meyberg et al., 2017). Another lateralized component which is commonly reported in studies of visual attention is the Late Directing Attention Positivity (LDAP) present from around 600 ms after cue onset over posterior electrode site as an enhanced positivity contralateral to the attentional cue direction. This component has been linked to attentional control processes in posterior parietal areas that are based on representations of visually mediated external space (c.f. Van Velzen et al., 2006). Importantly, we have recently identified a touch specific lateralized component present 200 ms before tactile target onset over central electrode sites (Gherri and Forster, 2012, Jones and Forster, 2012, Jones and Forster, 2013b). This Late Somatosensory Negativity (LSN) is characterised by an enhanced negativity contralateral to the side attention is oriented. The LSN has been suggested to reflect sensory specific preparatory processes prior to target onset as it is modulated by tactile target discriminability (Gherri et al., 2016), and, furthermore, it was shown to be diminished when attention is divided between vision and touch (Jones and Forster, 2013b). Thus, we hypothesised that this latter component may be modulated in somatoform disorder, if, as it has been suggested, the mechanisms underlying attentional orienting to the body are altered (c.f. Brown et al., 2010).

In addition to the neural correlates of attentional orienting during the cue target interval, electrophysiological data also allows to investigate attention effects on target processing. Attentional modulations are usually reported for mid and later latency components (e.g. Eimer and Forster, 2003, Jones and Forster, 2014 or for review Gomez-Raminez et al., 2016). These ERP effects have been shown to be sensitive to body posture (e.g. Eimer et al., 2001, Gherri and Forster, 2012), visibility of the body (Sambo, Gillmeister and Forster, 2009) and cognitive load (Jones and Forster, 2012, Jones and Forster, 2013b). If somatoform disorder is associated with how tactile targets are processed and selected, differences may be reflected in the timing or amplitude of attentional modulations and amplification on tactile target processing. Taken together, there are several established neural markers of focusing attention in the sense of touch and these can be used to assess whether attention may be altered in people reporting high numbers of somatic symptoms.

To investigate whether attentional orienting to the body or attentional selection of touch is changed in somatoform dissociation we first asked people to complete the Somatoform dissociation questionnaire (SDQ-20; Nijenhuis et al., 1996). The SDQ asks about different physical symptoms and body experiences during the past year. In particular, it focuses on conversion/dissociation of experiences (e.g., somatic or motor loss) rather than symptoms caused by minor physical illness or stress/depression based somatic symptoms. Two groups of participants were selected based on their score on the SDQ with one group reporting a high number of somatic symptoms while the control group reported no or a very low extent of somatic symptoms. These participants then completed an exogenous tactile attention task while concurrent EEG was recorded. Participants’ task was to discriminate tactile vibrations (targets) at the right or left hand while ignoring tactile taps (cues) applied prior to target presentations. Although the participants are instructed to ignore the cue, it automatically attracts attention, providing a measure of exogenous orienting. If somatoform dissociation is based on abnormal attentional orienting to the body, as induced by the cue, we expected group differences in the amplitude of lateralized attention components, particularly the LSN, in the cue target interval. However, if somatoform dissociation is based on abnormal tactile target selection and attentional amplification we expected to find group differences in attentional modulations of target processing. Therefore, this study provides evidence for the underlying neurocognitive attention changes associated with somatoform disorder.

## 2 Method

### 2.1 Participants

Participants completed an online version of the SDQ-20 questionnaire (Nijenhuis et al., 1996) until 18participants with a score ≥27 (“high group”) and 18participants with a score ≤21 (“low group”) were identified and invited to take part in the EEG part of the experiment. Due to technical difficulties two participants in the low group had to be replaced. In total, 125 participants completed the SDQ. For those invited to the EEG experiment, SDQ scores ranged between 27 and 50 in the high (mean score of 32.28) and between 20 and 21 in the low group (mean score of 20.25). Further, 26 were females (12 in the low and 14 in the high group) and 10 males (6 in the low and 4 in the high group) aged 18–52 years (overall mean age of 28 years with mean age of 29 for the low and 26 years for the high group). The study was approved by the Department of Psychology at City, University of London, ethics committee and all participants provided written informed consent. Participants were paid £8p/h for participation.

### 2.2 Stimuli and apparatus

MUS was assessed using the self-report Somatoform Dissociation Questionnaire (SDQ-20; Nijenhuis et al., 1996). The SDQ-20 was completed by participants online. Each item was rated on a 1 (“not at all”) to 5 (“extremely”) point Likert scale with total scores ranging from 20 to 100. A score of 20 indicates the absence of any somatoform disorder symptoms while a score around 30 on the SDQ-20 is taken as indication of somatoform dissociation. Respondents who rated an item as >1 were asked to indicate whether the cause of the symptom was known, and if so, to briefly explain it. Symptoms with a diagnosed medical explanation were treated as a score of 1 (“not at all”). However, it should be noted that it may be possible that the medical cause of the symptom is not known to the participant making it possible that some functional somatic symptoms with a medical explanation had been picked up. Participants invited to the EEG part of the study also completed the Body Awareness Questionnaire (Shields et al., 1989) which assesses the sensitivity to bodily rhythms, changes in normal function and anticipation of bodily reactions.

The EEG session took place at City, University of London in a dimly-lit, sound attenuated and electrically shielded room. Tactile stimuli were presented via 12-V solenoids (5 mm in diameter). The stimulators were attached to the external side of the right and left index finger using medical tape, and hands were placed 30 cm apart. To mask the sound made by the tactile stimulators, white noise (65 dB SPL) was played on two speakers located underneath the table throughout the task. Tactile cues consisted of a 50 ms single tap, while target stimuli lasted 300 ms with frequency of either 40 Hz or 200 Hz of touches resulting in two distinct vibratory sensations. Reponses were made verbally into a microphone, placed directly in front of the participant recording the onset of the response. Stimuli presentation and behavioural responses were recorded using E-prime version 2 (Psychology Software Tools, Inc.). EEG was recorded using a Brain Vision system and Brain Vision Recorder 2.1 software (Brain Products GmbH). A white fixation cross was presented throughout the experiment at the centre of a 19 inch monitor placed in front of the participant.

### 2.3 Design and procedure

**Fig. 1:**
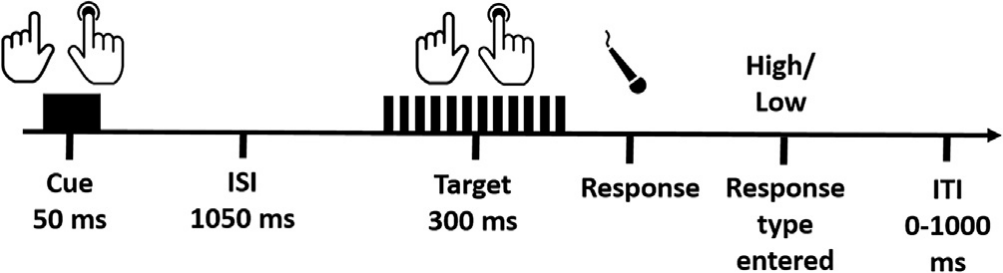
Schematic view of events in a trial. Each trial started with a cue (one tap) to the right or left hand followed by a fixed inter stimulus interval (ISI). The target (series of taps) was presented to the same (valid, as represented in the figure) or opposite hand (invalid) and was either a high or low frequency vibration. The participant responded by saying *High* or *Low* into a microphone. The vocal response onset was recorded and the experimenter then manually entered the response type on a keyboard in the adjacent room. The next trial started after a random inter-trial interval (ITI).

The main part of the study involved ten blocks of 40 trials each. In five of the blocks participants sat with their hand crossed over the midline, however, these trials were not further analysed. In each of the remaining 5 blocks participants sat with their hands in a normal, uncrossed position. In 20 trials the cue and target were presented to the same hand (“valid”) and in 20 trials the targets were presented to the opposite side to the cue (“invalid”). Valid and invalid trials were randomly intermixed. Each trial started with a 50 ms tactile cue, presented to the left or right hand (see Fig. 1). This was followed by a 1050 ms interval before a 40 Hz (low) or 200 Hz (high) target (300 ms) was presented to either the left or right hand. Participants were instructed to discriminate between target frequencies saying ‘high’ or ‘low’ as quickly as possible. Voice response onset was recoded with a microphone while response choice was keyed in by the experiment in the adjacent room. Once the response was entered, the next trial was initiated after a random inter-trial interval (ITI) of 0–1000 ms. Participants were informed at the start of the experiment to ignore the cue as it did not carry any information about the subsequent target location or frequency/type. Furthermore, before the start of the main experiment participants did one full experimental block as a practise block. Feedback on their performance (average speed and accuracy) was given at the end of each block.

### 2.4 Recording and analysis

Average response times for correct target discrimination and discrimination accuracy on valid and invalid trials were calculated. For each attention condition inverse efficiency (IE) scores were calculated [IE = reaction times/(1 − proportion of trials in which the wrong response was made)]. IE scores were subjected to mixed-factorial measures ANOVA with the within-subject factor Attention (valid vs invalid), and Group (high vs low) as between-subject factor.

Electrophysiological data was recorded using BrainAmp amplifiers (BrainProducts GmBH) with a built in high-pass filter of 0.06 Hz and 64 equally spaced, active electrodes (M10 arrangement, see www.easycap.de), referenced to the right earlobe. Horizontal electro-occulogram (HEOG) was recorded from the outer canthi of the eyes to detect eye movements. After recording, the EEG was digitally re-referenced to the average of the right and left earlobe. Filtering and artefact rejection procedures applied were in line with previous studies of exogenous attention (e.g. Jones and Forster, 2012). In particular, data were first filtered with a high cut off filter of 40 Hz. Trials with eye movements (voltage exceeding ±40 µV relative to baseline at HEOG electrodes) or with other artefacts (voltage exceeding ±80 µV relative to baseline at all electrodes) were removed prior to averaging. Separate analyses were conducted for the cue-target interval (CTI) and ERPs elicited by the tactile targets (post-target interval).

#### 2.4.1 CTI analysis

EEG was epoched offline from 100 ms before to 1100 ms after the cue onset. Baseline correction was performed to the 100 ms period preceding the onset of the cue. To investigate lateralised effects of attention, three time intervals were analysed; ADAN (400–600 ms), LDAN (600–800 ms) and LSN (900–1100 ms), in line with previous studies (Gherri et al., 2016, Jones and Forster, 2012). Each time interval was analysed separately with mixed-factors ANOVA including the between-subject factor Group (high vs low SDQ-20 score), and within-subject factors Region (Regions where the presence of lateralized ERP components has been previously reported: anterior, central vs posterior), Hemisphere (contralateral vs ipsilateral to the cue location) and Electrode (21/34, 37/49 vs 22/33 corresponding to F3/4, F7/8 vs FC5/6 for anterior regions; 17/11, 31/24 vs 47/39 corresponding to C3/4, CP5/6 vs T7/8 for central regions; and 44/42, 45/41 vs 29/26 corresponding to P3/4, P7/8, O1/2 for posterior regions).

#### 2.4.2 Post-target analysis

For post-target analysis, the EEG was epoched offline into 400 ms periods: 100 ms before and 300 ms after the target onset. Baseline correction was performed at the 100 ms period preceding onset of the target. Average amplitudes for time windows centred on the peak over the hemisphere contralateral to the target (averaged across all conditions) of the N80 and P100 (76–110 ms), N140 (112–148 ms), and Nd (150–250 ms). For each time interval mean amplitudes were analysed using a mixed-factors ANOVA with the between-subject factor Group (high vs low SDQ-20 score), and within-subject factors; Attention (valid vs. invalid cue), Hemisphere (ipsilateral vs. contralateral to target), Region (central, central-posterior versus central-temporal) and Electrode (7/3, 6/4 and 17/11 corresponding to FC1/2, CP1/2 and C3/4 for the central region, 16/12, 30/25 and 46/40 corresponding to CP3/4, CP5/6 and TP7/8 for the central-posterior region versus 32/23, 31/24 and 47/39 corresponding to FC5/6, C5/6 and T7/8 for the central-temporal region). As the aim of the study was to examine group difference in attention modulation and for brevity only significant results involving these factors are reported.

## 3 Results

### 3.1 Behavioural analyses

The high and low group scored similar on the BAQ (mean of 67 and 77, respectively; p = 0.22) indicating no difference in their body awareness. Both groups were highly accurate in the exogenous attention task (high group 95% correct; low group 97% correct) with the high group (mean of 661 ms) responding on average 44 ms faster than the low group (mean of 705 ms). To examine any behavioural differences between the high and low SDQ groups, inverse efficiency (IE) scores were calculated. The IE is the effect of cueing on mean response times while considering accuracy of target discrimination, thus controlling for any speed/accuracy trade-offs. ANOVA of IE scores on valid and invalid cued trials revealed a significant main attention effect (F(1, 34) = 7.24, *p* = .011, ƞ_p_^2^ = 0.176) with on average lower IE scores on valid (*M* = 684 ms*, SE* = 23) compared to invalid trials (*M* = 693 ms, *SE* = 22) but no main effect of Group or interaction between Group and Attention (all F(1, 34) ≤ 2.70, p ≥ 0.110, ƞ_p_^2^ ≥ 0.074). Taken together, these behavioural results show a clear attention effect with overall faster and more accurate responses to touch on previously cued locations.

### 3.2 Lateralized ERP components in the cue-target interval

**Fig. 2:**
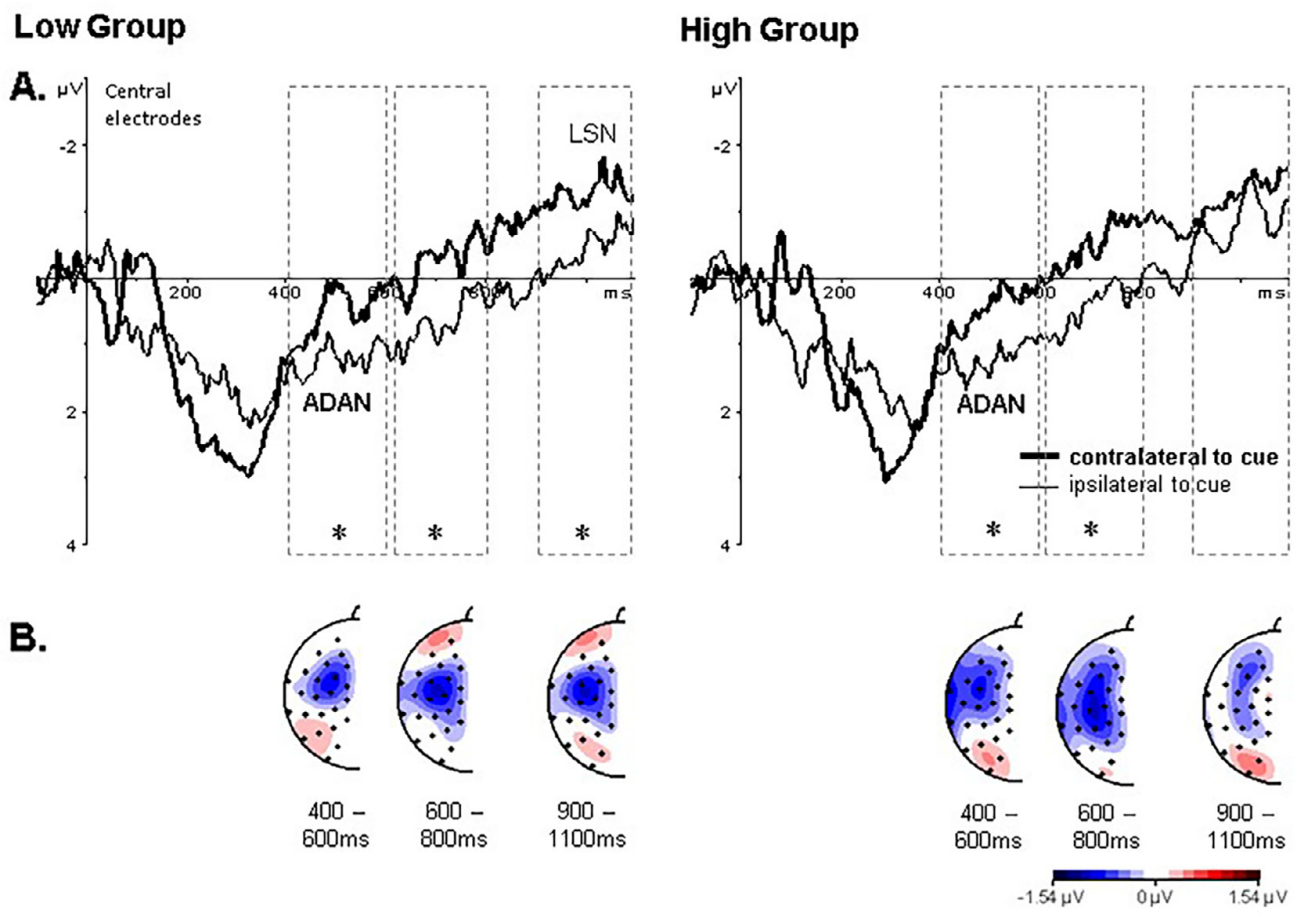
Cue-target ERPs separate for the low (left side) and high (right side) group: Panel A shows grand averaged ERPs responses to the onset of the cue pooled over central electrodes (corresponding to C3/4, CP5/6, T7/8) over somatosensory cortex contralateral (thick lines) and ipsilateral (thin lines) to the cue side. The dotted lines outline the statistical analyses windows with stars indicating statistical significance (p < 0.05) between the amplitudes over the contra- and ipsilateral hemispheres. The waveforms show the presence of the ADAN component for both groups 400–600 ms after cue onset, while the LSN is only reliably present in the low group in the last 200 ms of the cue-target interval. Panel B shows topographic maps of the time windows of lateralized ERP components; namely, the ADAN (400–600 ms), LDAP (600–800 ms) and LSN (900–1100 ms). These maps were generated by subtracting ERP waveforms elicited at electrodes ipsilateral to the cue from homologous electrodes contralateral to the cue.

Studies investigating attentional orienting have reported a succession of lateralized ERP components during the cue target interval, namely the ADAN, LDAP and LSN. We were particularly interested in modulations of the LSN present over central areas as this component has been linked to preparatory processes prior to tactile stimuli. Fig. 2a shows grand averaged ERPs to tactile cues ipsilateral and contralateral to the cued hand pooled over electrodes over the central region separate for the low and high groups (left and right graph, respectively). These graphs suggest that the LSN (i.e. the difference between activity contralateral and ipsilateral to the cue 200 ms before target onset) is diminished in the high group. In addition, Fig. 2b shows topographic maps of the difference of activity at homologous electrodes contralateral minus ipsilateral to the cue for the time range of the ADAN, LDAP and LSN separate for the low and high groups. These show the presence of the ADAN (left maps; 400–600 ms) over frontal central sites with an enhanced negativity contralateral to the cue side. This negativity continues for the following time window as no LDAP (middle maps; 600–800 ms), which would be shown by an enhanced positivity over posterior sites, is present. Finally, before the onset of the target the LSN (right maps; 900–1100 ms) is present with an enhanced negativity over central electrode sites; importantly, the strength of the LSN is diminished for the high group. Together, these observations suggest initial normal attentional orienting but a reduction in preparatory somatosensory attentional processes (i.e. LSN) in the high group.

These informal observations were scrutinised by statistical analysis; that is, ANOVAs with Region (anterior, central, posterior), Cue (left, right), Hemisphere (left, right) and Electrode Site (three electrode locations within each Region; see method section) as within-subject factors; and Group (low, high) as between-subject factor separate for the time windows of the lateralized ERP components (400–600 ms: ADAN, 600–800 ms: LDAP and 900–1100 ms: LSN) were conducted. The presence of lateralized ERP components is reflected in Hemisphere × Cue interactions. For each of the three analyses windows main effects of Electrode Site (all F(2,68) ≥ 6.89, p ≤ .003, ƞ_p_^2^ ≥ 0.154) and Region × Hemisphere × Cue interactions (all F(2,68) ≥ 5.66, p ≤ 0.005, ƞ_p_^2^ ≥ 0.143) were present suggesting topographic specificity of lateralized ERP components. In addition, for the 600–800 ms analysis window a Hemisphere × Cue interaction (F(1,34) = 8.81, p < 0.01, ƞ_p_^2^ = 0.21), and importantly, for the 900–1100 ms analysis window a Group × Hemisphere × Cue interaction (F(2,68) = 4.37, p < 0.05, ƞ_p_^2^ = 0.11 F(2,68) = 4.37, p < 0.05, ƞ_p_^2^ = 0.11) suggesting group differences were present in the lateralized ERP components prior to target onset.

The overall analyses showing interactions involving the factors Region, Hemisphere and Cue were followed up by separate analyses for the different Region for the first two analyses windows. The Group, Hemisphere by Cue interaction in the 900–1100 ms analysis window was followed up by separate analyses for each group. For the 400–600 ms and 600–800 ms analysis windows, significant Hemisphere × Cue interactions were present for anterior and central Regions (all F(1,34) ≥ 14.43, p ≤ 0.001, ƞ_p_^2^ ≥ 0.298) confirming the presence of the ADAN with enhanced negativity over frontal areas of the hemisphere contralateral to the cue direction (see Fig. 2). In line with out hypotheses for the 900–1100 ms analysis window, only for the low group significant Hemisphere × Cue interactions were present at anterior and central regions confirming the presence of the LSN (F(1,17) = 6.00, p = 0.025, ƞ_p_^2^ = 0.261 and F(1,17) = 8.43; p = 0.01, ƞ_p_^2^ = 0.337, respectively) while these interactions did not reach significance for the high group (F(1,17) = 3.24, p = .09, ƞ_p_^2^ = 0.160 and F(1,17) = 1.69; p = 0.21, ƞ_p_^2^ = 0.091, respectively). Taken together, these statistical analyses confirm the initial and sustained presence of the ADAN, reflecting general attentional orienting, over fronto-central regions in both groups. Importantly, and in line with our hypotheses, the subsequent LSN, signifying attentional orienting to the body, was present in the low group but was diminished in the high group.

### 3.3 Post-target ERP components

**Fig. 3:**
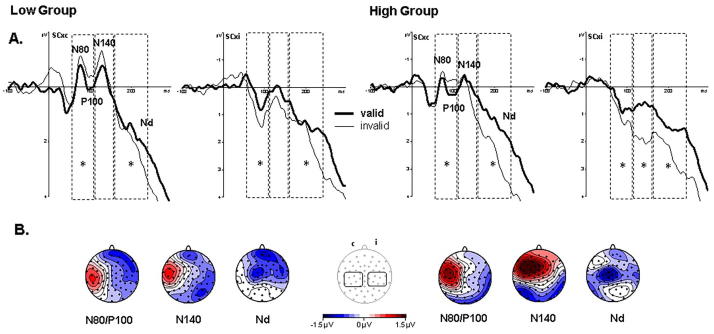
Post-target ERPs: Panel A shows grand averaged ERP responses to the onset of tactile target stimuli pooled over somatosensory cortex (SCx) electrodes contralateral (c) and ipsilateral (i) to the tactile target on valid (thick lines) and invalid (thin lines) cue trials separate for the low (left graphs) and high (right graphs) group. Dashed lines outline the statistical analyses windows with stars (*) indicating statistically significant (p < 0.05) attentional differences. Panel B shows topographic maps of the attention effects (ERPs on valid minus invalid cue trials) for the analysis time windows of the N80 and P100, the N140 and Nd components with the left side of the topographic maps showing amplitude distributions contralateral to the target stimulus. The centrally located electrode map outlines the electrodes used in the statistical analyses and their pooled amplitude changes over time are shown in panel A. These figures show reliable attention effects in the high group across all components and overall stronger attention effects seen in a wider topographic distribution of attentional differences.

Fig. 3a shows grand averaged ERP waveforms in response to target presentations at validly and invalidly cued locations separate for the low (left) and high (right) groups over somatosensory cortex contralateral to the target presentation. The graphs show similar attentional modulations for both groups for mid and late latency components over somatosensory areas. Furthermore, Fig. 3b shows topographic maps of the attention effects (i.e. ERPs on valid minus ERPs on invalid trials) for mid and late latency components (i.e. the time window of the N80/P100, N140 and Nd). These maps also show strong attentional modulations at these components which are enhanced for the high group. To confirm these observations ANOVAs with factors Attention (valid, invalid trials), Hemisphere (ipsilateral, contralateral to target location), Laterality (central, central-temporal, central-posterior) and Electrode (three electrode sites for each Laterality, see method section) and Group (low, high) were conducted for three time windows centred over the N80 and P100 components, the N140 component and the Nd. For clarity, only significant main effects and interactions of Attention and Group are reported, and, where appropriate, these are Greenhouse-Geisser corrected values.

For the analysis window spanning the N80 and P100 components a Hemisphere × Attention (F(1,34) = 40.27, p < 0.001, ƞ_p_^2^ = 0.543) and a Laterality × Hemisphere × Attention (F(2,68) = 7.04, p = 0.002, ƞ_p_^2^ = 0.171) interaction was present. Follow up analyses separate for the ipsi- and contralateral hemispheres showed a main effect of Attention (F(1,34) = 4.65, p = 0.038, ƞ_p_^2^ = 0.120) over the ipsilateral hemisphere while over the contralateral hemisphere Laterality × Attention (F(2,68) = 5.06, p = 0.015, ƞ_p_^2^ = 0.129) and Electrode × Attention (F(2,68) = 4.2, p = 0.026, ƞ_p_^2^ = 0.110) interactions were present. Follow up analyses showed a main effect of Attention over contralateral central-lateral and central-posterior areas (all F(1,34) > 5.09, p < 0.031, ƞ_p_^2^ > 0.130). Therefore, attentional modulations (difference between ERPs elicited on valid compared to invalid cue trials) were present at early to mid-latency ERP components over the hemisphere ipsi- and contralateral to the tactile stimulus side. These attentional modulations were comparable in both groups.

For the N140 component a Hemisphere × Attention (F(1,34) = 44.16, p < 0.001, ƞ_p_^2^ = 0.565), a Laterality × Hemisphere × Attention (F(2,68) = 8.5, p = 0.001, ƞ_p_^2^ *=* 0.2) and a Laterality × Hemisphere × Attention × Group (F(2,68) = 4.62, p = 0.013, ƞ_p_^2^ = 0.12) interaction were present. Follow up analyses separate for the factor Group showed a Hemisphere × Attention interaction for both groups (all F(1,34) > 14.34, p < 0.001, ƞ_p_^2^ > 0.458) and a Hemisphere × Laterality × Attention interaction for the low group (F(2,68) = 6.62, p = 0.005, ƞ_p_^2^ = 0.28). Follow up analysis separate for factor Hemisphere showed an Attention main effect over the ipsilateral hemisphere only for the high group (F(1,34) = 14.5, p = 0.001, ƞ_p_^2^ = 0.46). For the hemisphere contralateral to the target a Laterality × Attention interaction was present for the high group (F(1,34) = 4.15, p = 0.044, ƞ_p_^2^ = 0.196), while this interaction was only close to significance for the low group (F(1,34) = 3.12, p = 0.062, ƞ_p_^2^ = 0.155). Follow up analyses separate for the factor Laterality did not reveal a significant attention effect in the high group. Taken together, reliable mid-latency attention modulations were only present for the high group over the hemisphere ipsilateral to the target.

The following Nd analysis window showed the presence of a main effect of Attention (F(1,34) = 13.93, p = 0.001, ƞ_p_^2^ = 0*.*291), interactions of Attention with Laterality (F(2,68) = 12.68, p < 0.001, ƞ_p_^2^ *=* 0.271), and with Electrode (F(2,68) = 7.06, p = 0.004, ƞ_p_^2^ = 0.172) and a three way interaction between Attention × Laterality × Group (F(2,68) = 3.4, p = 0.042, ƞ_p_^2^ = 0.091). Follow up analyses separate for the Group factor showed a main effect of Attention for the high group (F(1,34) = 14.7, p = 0.001, ƞ_p_^2^ = 0*.*464), in addition to Attention × Electrode and Attention × Laterality interactions (all F(2,68) > 9.57, p ≤ 0.001, ƞ_p_^2^ ≥ 0.364). For the low group, an Attention × Hemisphere × Region (F(2,34) = 6.32, p = 0.006, ƞ_p_^2^ = 0.271) and an Attention × Hemisphere × Region × Electrode (F(4,68) = 3.15, p = 0.028, ƞ_p_^2^ = 0.152) interactions were present. Follow up analyses separate for the Hemisphere factor showed an Attention × Region × Electrode interaction only for the ipsilateral hemisphere (F(4,68) = 3.07, p = 0.037, ƞ_p_^2^ = 0.153). Further follow up analyses, separately for the factor Laterality confirmed a main effect of Attention (F(1,17) = 6.08, p = 0.025, ƞ_p_^2^ *=* 0.263) for the ipsilateral, posterior area only. These statistical results confirm the presence of attentional modulations at later latencies in both groups; however, these were stronger and more widespread for the high group.

## 4 Discussion

Previous research has suggested that changes in attention contribute to high somatic symptom reporting in the absence of a medical cause (Brown et al., 2010, Horvath et al., 1980, Roelofs, 2003). To investigate the neural basis of attentional changes we invited participants who scored high on the SDQ indicating the presence of a number of functional somatic symptoms and, in addition, we invited a control group who scored low on the SDQ indicating absence or very weak presence of somatic symptoms to perform a tactile exogenous attention task while their brain activity was recoded. On each trial they were presented with a tactile cue to one of their hands that they were instructed to ignore as it did not inform them about where (same of opposite hand) or what (high or low frequency) the target would be. Overall, participants were faster to discriminate tactile vibrations at the cued hand showing similar effects of attention on behavioural responses in the two groups. However, electro-cortical recordings revealed group differences in attention effects on somatosensory areas. In particular, we found that the LSN, a correlate of tactile attentional orienting to the body was diminished in people reporting high number of unexplained somatic symptoms. This suggests that the preparatory process for an upcoming tactile event is different in people with high SDQ scores. Further, attentional modulations of target processing were similar for both groups for early latencies; however, at mid and later latencies attentional modulations differed. These were present more strongly, in that these were prolonged, had enhanced amplitude and broader topographic spread in the group scoring high on the SDQ. Taken together, our results show that the brain processes during both orienting of attention and tactile stimulus processing are altered in people with high somatic symptom reporting.

A previous study by Brown et al. (2010) reported a greater behavioural attention effect in a group scoring high on the SDQ compared to a low group on an exogenous attention task. While we also found faster responses to targets at the previously cued location, this attention effect did not differ between groups. However, in contrast to our study Brown and colleagues employed several SOA and manipulated the emotional context. They reported a significant group difference in the attention effect following a neutral film but not a trauma film at a SOA of 1000 ms with diminished attention effects present in the low SDQ group. Therefore, while we replicated the attention effects of the high group, the low group may have been more sensitive to the manipulations of Brown et al.’s study and, thus, showed a reduced attention effect.

The main aim of this study was to reveal differences in the brain processes associated with attentional orienting and touch processing in people reporting a high number of functional somatic compared to a group with absence or very low occurrence of such symptoms. For this, we first analysed lateralized attention effects during the *cue-target* interval. Following cue presentation a series of lateralized ERP components are elicited that are taken to reflect the orienting of attention. The ADAN is usually present from around 400 ms after cue onset as an enhanced negativity over anterior electrodes contralateral to the cue direction. The onset of the ADAN has been shown to be independent of the length of the cue target interval (Van Velzen et al., 2002) while it is sensitive to how easy a cue can be interpreted (Jongen et al., 2007). The ADAN has been suggested to reflect a supramodal attention mechanism responsible for the encoding and selection of task-relevant locations in space (Eimer et al., 2002; but see Green and McDonald, 2006, Meyberg et al., 2017 for alternative accounts). While the above-mentioned studies all investigated endogenous attentional orienting, we have recently shown that the ADAN is also present during exogenous orienting of attention (Jones and Forster, 2012, Jones and Forster, 2013b). Likewise, in the current study the ADAN was present over anterior and central electrode sites with an enhanced negativity over electrodes contralateral to the cue from 400 ms after cue onset. Furthermore, the ADAN continued to be present as the LDAP was absent over posterior sites. Importantly and in line with our hypothesis, the ADAN did not differ for the high and low SDQ group, suggesting no group difference in the initial attentional location selection mechanisms.

In contrast to the ADAN, we expected a group difference in the presence of the LSN, a marker more closely linked to somatic sensory processing. In line with previous research from our group (Gherri and Forster, 2012, Gherri et al., 2016), the LSN was present 200 ms before target onset with an enhanced negativity over central and anterior electrodes contralateral to the cue. Although this component might appear as a continuation of the ADAN, it has been shown to be functionally different and independent of the ADAN, reflecting somatic rather than external coding of space (see Gherri and Forster, 2012 for an in-depth discussion). Importantly, we found that the magnitude of the LSN differs between the two groups; as the LSN is diminished in the high group while it is clearly present in the low group. It has previously been shown that the LSN is enhanced with increased tactile discrimination difficulty, (Gherri et al., 2016) while, on the other hand, it has been shown to be reduced when simultaneously engaging in a visual detection task during a tactile exogenous attention task (Jones and Forster, 2013b). Because tactile discrimination performance was high and did not behaviourally differ between groups, an effect of discrimination difficulty cannot account for the group difference we found. Thus, the absence of a reliable LSN in the high group suggests diminished ability to disengage and orient attention to a specific body part, in this case the hands. One should note that participants were instructed to ignore the cue; however, as indicted by both the presence of the ADAN following the cue and effects on behavioural responses, it is evident that these cues reflexively orient attention in both of our participant groups. Nevertheless, in the group with high functional somatic symptoms the electrophysiological marker of orienting attention to the body was diminished.

In addition to analysing the electro cortical response following the cue, we also analysed tactile *post-target* processing depending on whether these were presented at the previously cued hand or at the opposite hand. The attentional modulations of target processing we report here are in line with previous ERP studies of tactile exogenous attention. In particular, we found an early attentional modulation in the time range of the N80 and P100 components with an enhanced negativity in response to tactile stimuli following an invalid cue compared to targets at the validly cued location. Again, this indicates that even though the cue was to be ignored, it still had an effect on how the target was processed. This exogenous attentional modulation was also present at the mid-latency N140 component, but reached significance only in the high group over the hemisphere ipsilateral to the tactile target. Work from our lab has recently linked this early enhanced negativity to exogenous processing of the tactile cue (Jones and Forster, 2012, Jones and Forster, 2014), and has been shown to be delayed when engaging in another visual task (Jones and Forster, 2013a, Jones and Forster, 2013b). Interestingly, we found that this early exogenous effect is prolonged in the high group. Furthermore, while attentional modulations at later latencies, so called Nd, showed an enhanced negativity for tactile stimuli at validly cued locations was present in both groups, this later attention effect were stronger and more widespread in the high. Therefore, across different ERP components elicited by tactile targets we show increased attention effects on touch processing in our high compared to our control group.

Taken together, we provide electrophysiological evidence in line with the notion that changes in attention in people with high number of functional somatic symptoms. Importantly, in the current study only participants with non-clinical somatoform dissociation took part in an exogenous tactile attention experiment. Nevertheless, we could show that even in this non-clinical group a specific neural marker (i.e. the LSN) reflecting attentional orienting to the body is diminished. On the other hand, effects of attentional selection on touch processing are prolonged at mid-latency (N140) and enhanced at later latencies (Nd). Thus, in contrast to the diminished attention orienting effect in the cue target interval, we found amplified attentional modulations of tactile target processing. Both modulations are in line with the notion that attention processes are different in people with functional somatic symptoms. However, the pattern of electrophysiological effects of attention we report here does not easily map on previous cognitive models of MUS or models of functional somatic symptoms which either assume increased attention allocation (e.g. Barsky and Wyshak, 1990) or a failing of attentional filtering (e.g. Rief and Barsky, 2005).

Yet, this electrophysiological pattern may map onto recent predictive coding accounts of MUS and functional somatic symptoms (Edwards et al., 2012, Van den Bergh et al., 2017). In particular, Edwards et al. (2012) propose that ‘misdirection of attention’ leads to the formation of false priors or expectations, an account that may be reflected by our findings of the diminished ability to specifically orient to the relevant body part (i.e. absence of LSN). Such false priors may lead to increased prediction errors and increased attentional gain of target processing (Feldman and Friston, 2010). In line with this account, we found enhanced attentional modulations at mid and later latencies of target processing. Future research may directly test the relation between false priors and attentional modulations in people with MUS or functional somatic symptoms. Taken together, our results support the notion of changed attentional processes in MUS and functional somatic symptoms that are best explained by a predictive coding framework.

Finally, our findings are in line with recent therapeutic developments that have shown promising therapeutic gain through manipulating body focused attention such as physiotherapy based treatments for functional motor symptoms (e.g. Nielsen et al, 2017), CBT for dissociative seizures (e.g. Goldstein et al., 2003), and mindfulness for chronic pain management (e.g. Hilton et al., 2017).

## Conflict of interest

None of the authors have potential conflicts of interest to be disclosed.

## References

[b0005] American Psychiatric Association (2013). Diagnostic and statistical manual of mental disorders: (DSM-5®).

[b0010] Aronson K.R., Barrett L.F., Quigley K.S. (2001). Feeling your body or feeling badly: evidence for the limited validity of the Somatosensory Amplification Scale as an index of somatic sensitivity. J Psychosom Res..

[b0015] Barsky A.J., Borus J.F. (1995). Somatization and medicalization in the era of managed care. JAMA.

[b0020] Barsky A.J., Wyshak G. (1990). Hypochondriasis and somatosensory amplification. Br J Psychiatry.

[b0025] Bohmelt A.H., Nater U.M., Franke S., Hellhammer D.H., Ehlert U. (2005). Basal and stimulated hypothalamic-pituitary-adrenal axis activity in patients with functional gastrointestinal disorders and healthy controls. Psychosom Med.

[b0030] Brown R.J., Danquah A.N., Miles E., Holmes E., Poliakoff E. (2010). Attention to the body in nonclinical somatoform dissociation depends on emotional state. J Psychosom Res.

[b0035] Brown R.J., Poliakoff E., Kirkman M.A. (2007). Somatoform dissociation and somatosensory amplification are differentially associated with attention to the tactile modality following exposure to body-related stimuli. J Psychosomatic Res.

[b0040] Deary V., Chalder T., Sharpe M. (2007). The cognitive behavioural model of medically unexplained symptoms: a theoretical and empirical review. Clin Psychol Rev.

[b0045] Edwards M.J., Adams R.A., Brown H., Pareés I., Friston K.J. (2012). A Bayesian account of 'hysteria'. Brain.

[b0050] Eimer M., Cockburn D., Smedley B., Driver J. (2001). Cross-modal links in endogenous spatial attention are mediated by common external locations: evidence from event-related brain potentials. Exp Brain Res.

[b9000] Eimer M., Forster B. (2003). Modulations of early somatosensory ERP components by transient and sustained spatial attention. Experimental brain research.

[b0055] Eimer M., Forster B., Van Velzen J., Prabhu G. (2005). Covert manual response preparation triggers attentional shifts: ERP evidence for the premotor theory of attention. Neuropsychologia.

[b0060] Eimer M., Maravita A., Van Velzen J., Husain M., Driver J. (2002). The electrophysiology of tactile extinction: ERP correlates of unconscious somatosensory processing. Neuropsychologia.

[b0065] Feldman H., Friston K.J. (2010). Attention, uncertainty, and free-energy. Front Hum Neurosci.

[b0070] Forster B., Sambo C.F., Pavone E.F. (2009). ERP correlates of tactile spatial attention differ under intra- and intermodal conditions. Biol Psychol.

[b0075] Gherri E., Forster B. (2012). Crossing the hands disrupts tactile spatial attention but not motor attention: evidence from event-related potentials. Neuropsychologia.

[b0080] Gherri E., Gooray E., Forster B. (2016). Cue-locked lateralized components in a tactile spatial attention task: evidence for a functional dissociation between ADAN and LSN. Psychophysiology.

[b0085] Goldstein L.H., McAlpine M., Deale A., Toone B.K., Mellers J.D. (2003). Cognitive behaviour therapy with adults with intractable epilepsy and psychiatric co-morbidity: preliminary observations on changes in psychological state and seizure frequency. Behav Res Ther.

[b0090] Gomez-Ramirez M., Hysaj K., Niebur E. (2016). Neural mechanisms of selective attention in the somatosensory system. J Neurophysiol.

[b0095] Green J.J., McDonald J.J. (2006). An event-related potential study of supramodal attentional control and crossmodal attention effects. Psychophysiology.

[b0100] Hatcher S., Arroll B. (2008). Assessment and management of medically unexplained symptoms. BMJ..

[b0105] Hilton L., Hempel S., Ewing B.A., Apaydin E., Xenakis L., Newberry S., Colaiaco B., Maher A.R., Shanman R.M., Sorbero M.E., Maglione M.A. (2017). Mindfulness meditation for chronic pain: systematic review and meta-analysis. Ann Behav Med.

[b0110] Hopf J.M., Mangun G.R. (2000). Shifting visual attention in space: an electrophysiological analysis using high spatial resolution mapping. Clin Neurophysiol.

[b0115] Horvath T., Friedman J., Meares R. (1980). Attention in hysteria: a study of Janet's hypothesis by means of habituation and arousal measures. Am J Psychiatry.

[b0120] Jones A., Forster B. (2013). Independent effects of endogenous and exogenous attention in touch. Somatosens Mot Res.

[b0125] Jones A., Forster B. (2013). Lost in vision: ERP correlates of exogenous tactile attention when engaging in a visualtask. Neuropsychologia.

[b0130] Jones A., Forster B. (2014). Neural correlates of endogenous attention, exogenous attention and inhibition of return in touch. Eur J Neurosci.

[b0135] Jones A., Forster B. (2012). Reflexive attention in touch: an investigation of event related potentials and behavioural responses. Biol Psychol.

[b0140] Jongen E.M., Smulders F.T., Van der Heiden J.S. (2007). Lateralized ERP components related to spatial orienting: discriminating the direction of attention from processing sensory aspects of the cue. Psychophysiology.

[b0145] Kienle J., Rockstroh B., Bohus M., Fiess J., Huffziger S., Steffen-Klatt A. (2017). Somatoform dissociation and posttraumatic stress syndrome–two sides of the same medal? A comparison of symptom profiles, trauma history and altered affect regulation between patients with functional neurological symptoms and patients with PTSD. BMC Psychiatry.

[b0150] Mailloux J., Brener J. (2002). Somatosensory amplification and its relationship to heartbeat detection ability. Psychosom Med.

[b0155] Meyberg S., Sommer W., Dimigen O. (2017). How microsaccades relate to lateralized ERP components of spatial attention: a co-registration study. Neuropsychologia.

[b0165] Nielsen G., Buszewicz M., Stevenson F., Hunter R., Holt K., Dudziec M., Ricciardi L., Marsden J., Joyce E., Edwards M.J. (2017). Randomised feasibility study of physiotherapy for patients with functional motor symptoms. J Neurol Neurosurg Psychiatry.

[b0170] Nijenhuis E.R. (2004). Somatoform dissociation: phenomena, measurement, and theoretical issues.

[b0175] Nijenhuis E.R., Spinhoven P., Van Dyck R., Van der Hart O., Vanderlinden J. (1996). The development and psychometric characteristics of the Somatoform Dissociation Questionnaire (SDQ-20). J Nerv Ment Dis.

[b0180] Nobre A.C., Sebestyen G.N., Miniussi C. (2000). The dynamics of shifting visuospatial attention revealed by event-related potentials. Neuropsychologia.

[b0185] Posner M.I. (2016). Orienting of attention: then and now. Q J Exp Psychol.

[b0190] Ratcliffe N., Newport R. (2016). Evidence that subclinical somatoform dissociation is not characterised by heightened awareness of proprioceptive signals. Cogn Neuropsychiatry.

[b0195] Rief W., Barsky A.J. (2005). Psychobiological perspectives on somatoform disorders. Psychoneuroendocrinology.

[b0200] Rief W., Hiller W., Margraf J. (1998). Cognitive aspects of hypochondriasis and the somatization syndrome. J Abnorm Psychol.

[b0205] Rief W., Pilger F., Ihle D., Verkerk R., Scharpe S., Maes M. (2004). Psychobiological aspects of somatoform disorders: contributions of monoaminergic transmitter systems. Neuropsychobiology.

[b0210] Roelofs A. (2003). Goal-referenced selection of verbal action: modelling attentional control in the Stroop task. Psychol Rev.

[b0215] Sambo C.F., Gillmeister H., Forster B. (2009). Viewing the body modulates neural mechanisms underlying sustained spatial attention in touch. Eur J Neurosci.

[b0220] Shields S.A., Mallory M.E., Simon A. (1989). The body awareness questionnaire: reliability and validity. J Pers Assess.

[b0230] Van den Bergh O., Witthoft M., Petersen S., Brown R.J. (2017). Symptoms and the body: taking the inferential leap. Neurosci Biobehav Rev.

[b0235] Van Velzen J., Eardley A.F., Forster B., Eimer M. (2006). Shifts of attention in the early blind: an ERP study of attentional control processes in the absence of visual spatial information. Neuropsychologia.

[b0240] Van Velzen J., Forster B., Eimer M. (2002). Temporal dynamics of lateralized ERP components elicited during endogenous attentional shifts to relevant tactile events. Psychophysiology.

